# P49 The Barbara Ansell National Network for Adolescent Rheumatology – 10 years on

**DOI:** 10.1093/rap/rkac067.049

**Published:** 2022-09-28

**Authors:** Sarah Yorke, Victoria Harbottle, Flora McErlane, Rachel Tattersall, Janet McDonagh

**Affiliations:** Versus Arthritis, Chesterfield, United Kingdom; University of Newcastle, Newcastle upon Tyne, United Kingdom; Great Northern Children's Hospital, Newcastle upon Tyne, United Kingdom; Sheffield Children's Hospital NHS Trust, Sheffield, United Kingdom; Sheffield Teaching Hospitals NHS Trust, Sheffield, United Kingdom; University of Manchester, Manchester, United Kingdom; NIHR Biomedical Research Centre, Manchester, United Kingdom; Royal Manchester Children's Hospital, Manchester, United Kingdom

## Abstract

**Introduction/Background:**

Over the last 10 years since BANNAR was established, there has been increasing awareness of the specific age and developmental needs of adolescents and young adults (AYA) with rheumatic disease. The aim of this study was to describe the current BANNAR membership to ensure representativeness and determine clinical and research capacity to inform future strategies.

**Description/Method:**

An online survey on Microsoft Forms (version 2021) was developed, piloted with the BANNAR leadership team and then disseminated to existing members in addition to any new members as part of the membership process.

**Discussion/Results:**

As of May 2022, there are 118 members. The BANNAR membership includes a wide variety of roles, from healthcare professionals reflecting the multidisciplinary team to charity representatives, academics, and researchers. Of the clinically active professionals (n = 93) there are more members working in the paediatric (n = 57) compared to the adult care setting (n = 34). There are 15 members in training posts, 16 members in research-only roles, and 9 charity representatives. Membership is predominantly female with a gender ratio of members F104: M14.

Members are predominantly White British (n = 81, 69%) and include representation from all regions of England and the devolved nations in addition to 1 researcher based in Australia. 27/116 (23.3%) members reported being currently involved in AYA research with 2 non-respondents. This research was wide ranging and involved both basic science as well as clinical research. Of the NHS clinicians (excluding the clinical academics), 34/79 (43%) had research in their job description and/or job plan.

Of 56 paediatric clinicians, 34 had access (61%) to a dedicated adolescent clinic in the paediatric setting. 37/93 NHS clinicians (40%) had access to a young adult clinic in adult services.

Of the 93 clinically active members, only 15 (16%) members reported regular in-service training for team members in AYA topics. 23/93 (25%) clinician members used/signposted team members to the Adolescent Health e-learning project (www.e-lfh.org.uk) but 55/93 (59%) reported that they were unaware of this resource.

**Key learning points/Conclusion:**

BANNAR is a national network with membership reflecting the multidisciplinary team in addition to researchers and charity representatives. As such, along with its youth advisory panel – Your Rheum – it is an excellent resource to support future AYA rheumatology research. However, there remain challenges for clinicians in contributing to research, with less than half having formal provision for research in their job descriptions. Furthermore, even in this committed group of AYA-focussed individuals, there was not universal access to adolescent and/or young adult clinics and there are still areas for development in AYA rheumatology training.

